# Crystal Structure of an SSB Protein from *Salmonella enterica* and Its Inhibition by Flavanonol Taxifolin

**DOI:** 10.3390/ijms23084399

**Published:** 2022-04-15

**Authors:** En-Shyh Lin, Yen-Hua Huang, Ren-Hong Luo, Zarrin Basharat, Cheng-Yang Huang

**Affiliations:** 1 Department of Beauty Science, National Taichung University of Science and Technology, No. 193, Sec.1, San min Rd., Taichung City 403, Taiwan; eslin7620@gmail.com; 2 Department of Biomedical Sciences, Chung Shan Medical University, No. 110, Sec.1, Chien-Kuo N. Rd., Taichung City 402, Taiwan; cicilovev6@gmail.com (Y.-H.H.); hong755225@gmail.com (R.-H.L.); 3 Jamil–ur–Rahman Center for Genome Research, Dr. Panjwani Center for Molecular Medicine and Drug Research, International Center for Chemical and Biological Sciences, University of Karachi, Karachi 75270, Pakistan; zarrin.iiui@gmail.com; 4 Department of Medical Research, Chung Shan Medical University Hospital, No. 110, Sec.1, Chien-Kuo N. Rd., Taichung City 402, Taiwan

**Keywords:** SSB, OB fold, taxifolin, dihydroquercetin, myricetin, quercetin, flavanonol, flavonol, PriB, *Salmonella enterica*

## Abstract

Single-stranded DNA (ssDNA)-binding proteins (SSBs) play a central role in cells by participating in DNA metabolism, including replication, repair, recombination, and replication fork restart. SSBs are essential for cell survival and thus an attractive target for potential anti-pathogen chemotherapy. In this study, we determined the crystal structure and examined the size of the ssDNA-binding site of an SSB from *Salmonella enterica* serovar Typhimurium LT2 (SeSSB), a ubiquitous opportunistic pathogen which is highly resistant to antibiotics. The crystal structure was solved at a resolution of 2.8 Å (PDB ID 7F25), indicating that the SeSSB monomer possesses an oligonucleotide/oligosaccharide-binding (OB) fold domain at its N-terminus and a flexible tail at its C-terminus. The core of the OB-fold in the SeSSB is made of a six-stranded β-barrel capped by an α-helix. The crystal structure of the SeSSB contained two monomers per asymmetric unit, which may indicate the formation of a dimer. However, the gel-filtration chromatography analysis showed that the SeSSB forms a tetramer in solution. Through an electrophoretic mobility shift analysis, we characterized the stoichiometry of the SeSSB complexed with a series of ssDNA dA homopolymers, and the size of the ssDNA-binding site was determined to be around 22 nt. We also found the flavanonol taxifolin, also known as dihydroquercetin, capable of inhibiting the ssDNA-binding activity of the SeSSB. Thus, this result extended the SSB interactome to include taxifolin, a natural product with a wide range of promising pharmacological activities.

## 1. Introduction

Single-stranded DNA (ssDNA)-binding proteins (SSBs) play a central role in cells by participating in DNA metabolism, including in replication, repair, recombination, and replication fork restart [1]. During these reactions, SSBs are required to maintain the transient unwinding of duplex DNA in the single-stranded state. SSBs bind tightly and cooperatively to ssDNA [2], regardless of sequence, and prevent premature annealing and unwanted nuclease digestion [3]. SSBs were formerly known as the DNA-unwinding proteins because of their ability to destabilize a DNA double helix [4]. Most, but not all, bacterial SSBs are active as homotetramers, in which four oligonucleotide/oligosaccharide-binding (OB) folds [5,6] form a DNA-binding domain [7,8,9,10]. The OB fold [11] in bacterial SSBs typically possesses a five-stranded β-barrel capped by an α-helix [12,13,14,15]. However, an additional strand (β6) is also found in some bacterial SSBs [10,16,17,18]. Additional β6 strands clamp two neighboring subunits together in a tetrameric SSB. Thus, SSBs from different organisms may exhibit different protein–DNA and protein–protein interaction specificities [18].

The eukaryotic equivalent of bacterial SSBs is replication protein A (RPA) [19]. Although bacterial SSBs [20] and RPA [19] share basic mechanistic functioning, they are different in terms of structure and many other functions [15,17,21,22,23,24]. For example, the canonical RPA is active as a heterotrimer composed of three subunits (RPA1, RPA2, and RPA3). Given the significant differences between RPA and bacterial SSBs, the pharmacological inhibition of bacterial SSBs may be used to target pathogens. The knowledge of the structure and of how bacterial SSBs can be inhibited is an advantage for the development of inhibitors.

*Salmonella enterica* is a common foodborne illness both in the United States and globally [25]. Clinically, salmonellosis may be manifested as gastroenteritis, septicemia, or enteric fever, and causes over 200,000 deaths and 22 million illnesses per year [26]. Currently, antibiotic-resistant *salmonella* strains are being reported at an alarming rate [27]. These multidrug-resistant *S. enterica* are spreading rapidly worldwide and can become untreatable [28,29,30]. Therefore, developing more useful antibiotics and identifying new targets in this pathogen are urgently needed to fight the growing threat of drug-resistant *S. enterica*. SSBs are essential for DNA replication and cell survival and, thus, are an attractive target for potential antipathogen chemotherapy [13,31,32,33]. *S. enterica* has six subspecies, and each subspecies has associated serovars that differ by antigenic specificity. *S. enterica* has over 2500 serovars; however, there are still no SSB structures from *S. enterica* available in the Protein Data Bank (PDB) for drug development. The structure of the *S. enterica* SSB (SeSSB) is needed as a molecular basis to formulate any inhibition model. Therefore, it is worth determining the crystal structure of the SeSSB.

Taxifolin (5,7,3′,4′-flavan-on-ol), also known as dihydroquercetin, belongs to the subclass flavanonols in the flavonoids, which in turn are a class of polyphenols [34]. Many polyphenols [35,36,37] can be developed as drug candidates [38,39] from the active confirmation of in vitro screens or in vivo evaluations [40]. Flavonoids are a family of polyphenolic compounds that are widespread in nature and are consumed as part of the human diet in significant amounts. Over 5000 different flavonoids have been identified, many of which display structure-dependent biological and pharmacological activities [41,42,43], including antimicrobial agents [44,45]. In addition to its use in antimicrobial infections, taxifolin also shows promising pharmacological activities in the management of inflammation, tumors, oxidative stress, and cardiovascular and liver disorders [34]. Results from the pharmacokinetics and safety profile analysis of taxifolin suggest the development of a drug for human use [46]. Taxifolin also enhances the efficacy of the antibiotics levofloxacin and ceftazidime in vitro, which have potential for the combinatory therapy of patients infected with methicillin-resistant *Staphylococcus aureus* [47]. Prior to this study, the effect of taxifolin on SSBs was unknown and should be elucidated.

*S. enterica* serovar Typhimurium is a leading cause of human gastroenteritis [25]. The incidence of non-typhoid salmonellosis is increasing worldwide, causing millions of infections and many deaths in the human population each year [25]. In this study, we determined the crystal structure and examined the size of the ssDNA-binding site of an SSB from *S. enterica* serovar Typhimurium LT2. We also identified that taxifolin could inhibit the ssDNA-binding activity of the SeSSB. Based on the structural comparison, the binding mode of taxifolin to the SeSSB is discussed and proposed.

## 2. Results

### 2.1. Cloning, Expression, Purification, Crystallization, and Data Collection of SeSSB

Based on the complete genome sequence of *S. enterica* serovar Typhimurium LT2 [25], the plasmid for SeSSB (*STM_4256*) expression was constructed [17,48,49]. This His-tagged protein was overexpressed in *Escherichia coli* and purified from the soluble supernatant by Ni^2+^-affinity chromatography. Approximately 10 mg of purified SeSSB was obtained from 1 L of an *E. coli* cell culture. Commercially available screens from Hampton research and Jena biosciences were employed for the crystallization trials. Crystals of the SeSSB were grown at room temperature by hanging drop vapor diffusion in 15% PEG400 and 100 mM MES at pH 6.5. The completeness was over 99% (Table 1).

### 2.2. Crystal Structure of the SeSSB

**Figure 1:**
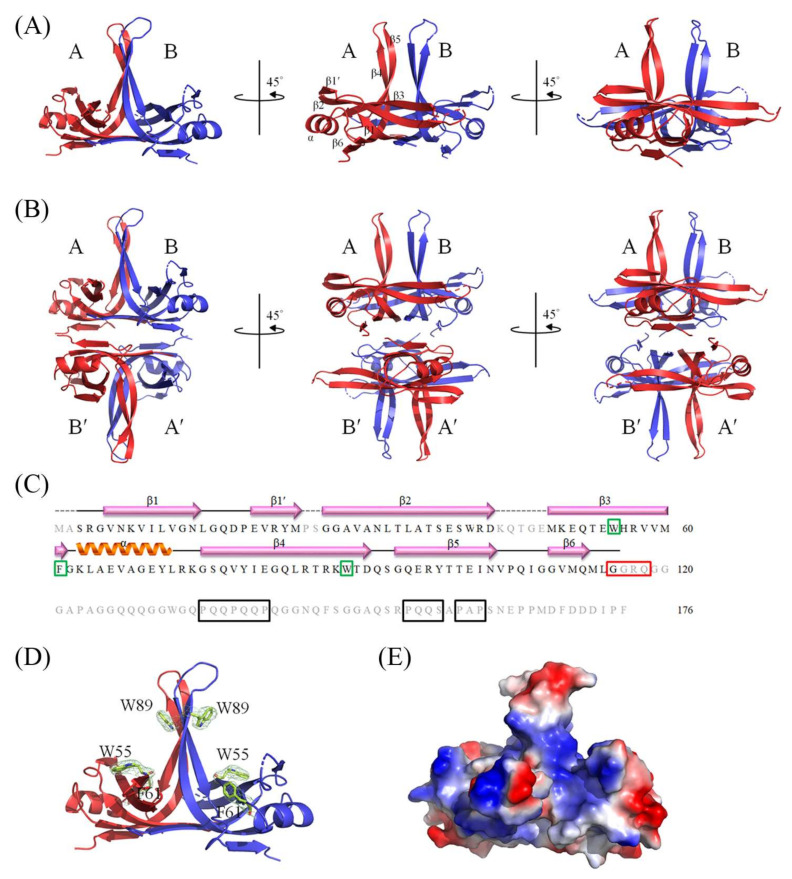
An SSB protein from *S. enterica* serovar Typhimurium LT2. (**A**) Crystal structure of SeSSB. Two monomers of the SeSSB were found per asymmetric unit. The core of the OB-fold in the SeSSB is made of a six-stranded β-barrel capped by an α-helix. The C-terminal region (residues 116–176) in the structure of the SeSSB was dynamic and unobserved. (**B**) The crystallographically related tetramer. Subunits A’ and B’ are symmetry-related molecules. Given that the oligomerization state of bacterial SSBs in solution is tetrameric, the crystallographically related tetramer A-B-A′-B′ is also shown. (**C**) Sequence of SeSSB. The secondary structural elements of the SeSSB are shown with the sequence. Residues colored in gray were not observed in the structure of the SeSSB. The putative PXXP motifs in the SeSSB are boxed in black. The GGRQ motif is boxed in red. The aromatic residues crucial for ssDNA binding are boxed in green. (**D**) The essential aromatic residues in SeSSB. These residues are conserved as F/Y/W in most SSB families. The corresponding residues in the SeSSB are W55, F61, and W89. The composite omit map (at 1.0 *σ*) showed the electron density of these aromatic residues in the SeSSB. (**E**) The electrostatic potential surface of SeSSB. The SeSSB contained many positively charged residues on the protein surface that may serve as a potential ssDNA-binding pocket.

The SeSSB structure was determined at a resolution of 2.8 Å (Table 1). The crystal of the SeSSB belonged to the space group P3_2_21 with cell dimensions of *a* = 91.89, *b* = 91.89, and *c* = 61.05 Å. The crystal structure of the SeSSB (PDB ID 7F25) was solved with the molecular replacement using the *E. coli* SSB (EcSSB) as a model (PDB ID 1EYG). Two monomers (subunit A and B) of the SeSSB were found in the asymmetric unit (Figure 1A). Given that the oligomerization state of bacterial SSBs in solution is tetrameric, the crystallographically related tetramer A-B-A′-B′ is also shown (Figure 1B). Namely, subunits A’ and B’ are symmetry-related molecules. In both of the subunits, only the N-terminal ssDNA-binding domain (residues 1–115) was ordered and observed. The C-terminal region (residues 116–176) in the structure of the SeSSB was dynamic, which is similar to the case in the EcSSB. Even in the N-terminal domain, residues in the loops L_12_ (residues 25–26) and L_23_ (residues 44–48) were disordered and unobserved (Figure 1C). The global architecture of the SeSSB monomer revealed an OB-fold structure. The core of the OB-fold in the SeSSB is made of a six-stranded β-barrel capped by an α-helix (Figure 1A). The β6 strand in the SeSSB is not found in some bacterial SSBs, such as *Streptomyces coelicolor* SsbB (ScSsbB) [18] and *Staphylococcus aureus* SsbA (SaSsbA) [15], SsbB (SaSsbB) [14], and SsbC (SaSsbC) [13]. According to the structural analysis, we noted that most of these SSBs without the β6 strand are from Gram-positive bacteria. The β6 strands in a tetrameric SSB have been proposed to be involved in exhibiting different protein–DNA and protein–protein interaction specificities among different SSBs [18]. The GGRQ motif, proposed as a regulatory switch for ssDNA binding [16], and the PXXP motifs, known to mediate the protein–protein interactions [50], were disordered and disappeared in our crystal structure (Figure 1C). In the EcSSB, the PXXP motifs occur at residues 139 (PQQP), 156 (PQQS), and 161 (PAAP) [50]. We noted that the PXXP motifs in the SeSSB were different. In the SeSSB, the first motif is duplicated to PQQPQQP while the third motif is shortened to PAP instead of PAAP in the EcSSB (Figure 1C). In the EcSSB–ssDNA complex, three essential aromatic residues, namely, W54, F60, and W88, participated in ssDNA binding via stacking interactions [10]. These residues are conserved as F/Y/W in most SSB families. The corresponding residues in the SeSSB are W55, F61, and W89 (Figure 1D), which may play a similar role to that of EcSSB in ssDNA binding. The SeSSB contained many positively charged residues on the protein surface that may serve as a potential ssDNA-binding pocket (Figure 1E).

### 2.3. Oligomeric State of SeSSB in Solution

**Figure 2:**
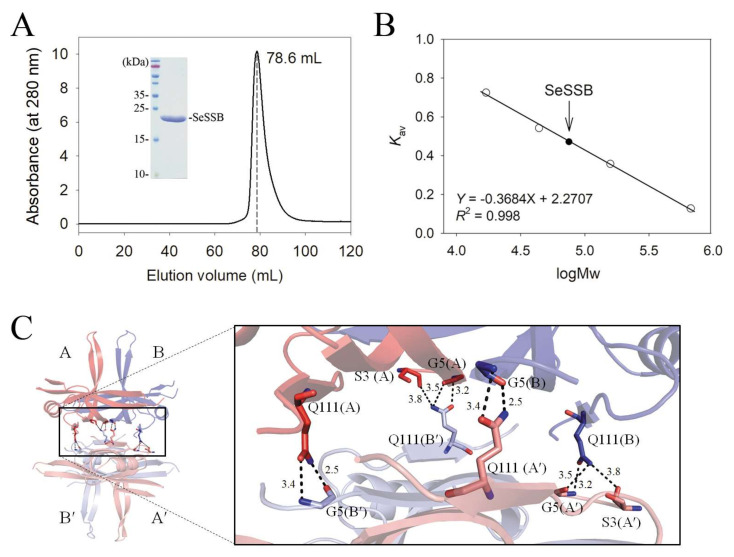
Oligomeric state of SeSSB. (**A**) Gel-filtration chromatographic analysis of the purified SeSSB. The corresponding single peak shows the eluting SeSSB. Coomassie Blue-stained SDS-PAGE (15%) of the purified SeSSB is also shown. (**B**) Native molecular mass of SeSSB. The native molecular mass of the SeSSB was estimated to be 76641 Da. The native molecular mass for SeSSB was approximately four times the mass of the monomer (~19 kDa) and therefore the SeSSB was a tetramer. (**C**) Structural analysis of the dimer–dimer interface of SeSSB. The structure of the SeSSB (the crystallographically related tetramer A-B-A′-B′) was used to explain how the tetramer forms. Hydrogen bonds and salt bridges were formed at the dimer–dimer interface of the SeSSB: S3(A)–Q111(B′), G5(A)–Q111(B′), G5(B)–Q111(A′), Q111(A)–G5(B′), Q111(B)–S3(A′), and Q111(B)–G5(A′). These residues from the subunit A and B are labeled in red and blue, respectively. The distance (Å) of the residues is also shown.

PriB, a kind of SSB, shares structural similarity with SSBs but is a dimeric protein with two OB folds [51,52,53,54,55]. We crystallized the SeSSB and determined its structure at pH 6.5; two monomers were shown per asymmetric unit (Figure 1A). We then attempted to confirm whether the oligomeric state of the SeSSB remains as tetramers at pH 6.5 (Figure 2). Through gel-filtration chromatography, the analysis of the purified SeSSB (5 mg/mL) using a Superdex 200 prep-grade column revealed a single peak with an elution volume of 78.6 mL (Figure 2A). As compared to the standard proteins and calculated from a standard linear regression equation, the native molecular mass of the SeSSB was estimated to be 76641 Da (Figure 2B). Based on the protein sequence, the predicted SeSSB monomer protein has a length of 176 amino acid residues and a molecular mass of ~19 kDa. The native molecular mass for SeSSB was approximately four times the mass of the monomer and therefore the SeSSB was a stable tetramer. Although the secondary structural element and overall architecture of the PriB monomer are similar to those of the SeSSB, we ruled out the possibility that SeSSB forms a dimer at pH 6.5.

The structure of the SeSSB (the crystallographically related tetramer A-B-A′-B′) was used to explain how the tetramer forms (Figure 2C). Hydrogen bonds and salt bridges were formed at the dimer–dimer interface of the SeSSB: S3(A)–Q111(B′), G5(A)–Q111(B′), G5(B)–Q111(A′), Q111(A)–G5(B′), Q111(B)–S3(A′), and Q111(B)–G5(A′). These residues were also conserved, such as in the EcSSB [50], *Klebsiella pneumonia* SSB (KpSSB) [56], and *Pseudomonas aeruginosa* SSB (PaSSB) [57]. However, residues S3 and G5 in SeSSB for tetramer formation are not found in *S. aureus* paralogous SSBs, namely, SaSsbA [15], SaSsbB [14], and SaSsbC [13]. Accordingly, we concluded that their tetramer formation mechanisms are different.

### 2.4. Binding of SeSSB to ssDNA of Different Lengths

**Figure 3:**
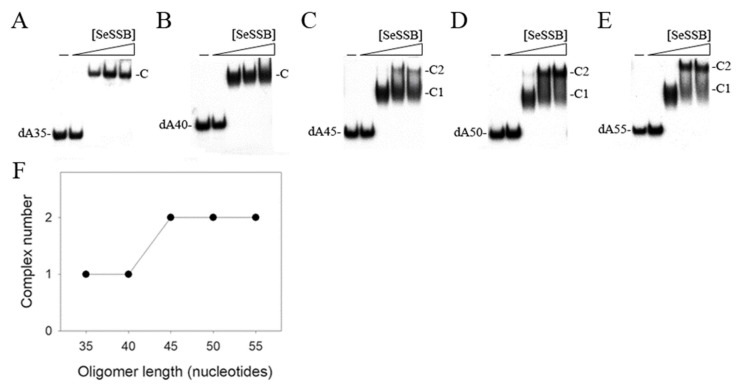
EMSA of SeSSB. The SeSSB protein (0, 500, 1000, 2000, and 4000 nM) was incubated at 25 °C with 1.7 nM of (**A**) dA35, (**B**) dA40, (**C**) dA45, (**D**) dA50, or (**E**) dA55. The resulting samples were resolved on a native 8% polyacrylamide gel at 4 °C in TBE buffer (89 mM Tris borate and 1 mM EDTA) for 1 h (for analyzing dA35 and dA40) or 1.5 h (for dA45–55) at 100 V and visualized by autoradiography. (**F**) Summary of the complex number of the SeSSB.

The binding of SSBs to ssDNA is independent of the sequence of DNA [3]. We studied the binding of SeSSB to ssDNA of different lengths with different protein concentrations using electrophoretic mobility shift analysis (EMSA). EMSA is a well-established approach in studies of molecular biology, allowing the detection of the distinct protein–DNA complex(es) [58]. The expected result of EMSA is that when the length of the nucleotides is sufficient for the binding of two or more protein molecules, the electrophoretic mobility of the higher SSB oligomer complex will be lower than that of the smaller protein oligomer complex. By using a series of ssDNA dT homopolymers (deoxythymidine oligonucleotide), the sizes of the binding site of SeSSB, KpSSB, and PaSSB were determined to be around 22 [49], 26 [56], and 29 nt [57], respectively. In addition, His-tagged and untagged SSBs have similar ssDNA-binding-site sizes [48]. In this study, dA homopolymers (deoxyadenosine oligonucleotide) were used to determine the binding-site size of the SeSSB and also to investigate the possible base preference of the SeSSB. The binding of the SeSSB to ssDNA dA homopolymers (dA35–55) was analyzed (Figure 3). We found that the binding patterns of the SeSSB to these dA homopolymers were similar to the dT homopolymers [48,49]. As observed from the EMSA, a single band shift was produced when the SeSSB was incubated with dA35 (Figure 3A) and dA40 (Figure 3B). Two different complexes with dA45 were observed for higher concentrations of SeSSB (Figure 3C). Although dA45 is only 5 nt longer than dA40, the pattern of the SeSSB–ssDNA complexes was very different. The presence of an extra 5 nt in dA45, as compared with dA40, provided enough interaction space for the binding of a second SeSSB molecule, i.e., one SeSSB occupied around 22 (45/2 = 22.5) nt ssDNA on average. Two distinct complexes were also observed for SeSSB binding to dA50 (Figure 3D) and dA55 (Figure 3E). Taking the results in this study (Figure 3F) and those of previous works together, the length of dA and dT ssDNA [49] for efficient binding of the SeSSB was approximately 22 nt.

### 2.5. Inhibition of the ssDNA-Binding Activity of SeSSB by Taxifolin

**Figure 4:**
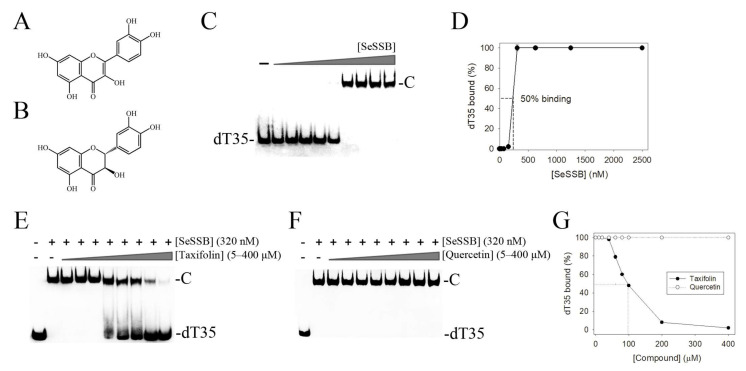
Inhibition of the ssDNA binding activity of the SeSSB by flavanonol taxifolin. (**A**) Molecular structure of the flavonol quercetin. (**B**) Molecular structure of the flavanonol taxifolin. (**C**) Binding of the SeSSB to ssDNA dT35. Purified SeSSB (0, 10, 19, 37, 77, 155, 310, 630, 1250, and 2500 nM) was incubated with biotin-labeled dT35 at 37 °C for 60 min. A total of 310 nM SeSSB was sufficient to reach 100% binding of the dT35. (**D**) The titration curve for determining the binding constant. The dissociation constant of the SeSSB to bind ssDNA dT35 was calculated to be 230 ± 20 nM. (**E**) Inhibition of SeSSB by taxifolin. SeSSB (320 nM) was incubated with taxifolin (0, 5, 20, 40, 60, 80, 100, 200, and 400 μM). (**F**) Inhibition test of SeSSB by quercetin. SeSSB (320 nM) was incubated with quercetin (5–400 μM). These polyphenol compounds were dissolved in 10% dimethyl sulfoxide. (**G**) An IC_50_ determination for SeSSB. Taxifolin could inhibit SeSSB binding to dT35, while quercetin could not. The IC_50_ value of the SeSSB for taxifolin was determined to be 98 ± 12 μM.

The flavonol quercetin can bind to PaSSB but cannot inhibit the binding activity of PaSSB [31]. In this study, we attempted to use the quercetin analogue, i.e., taxifolin (DrugBank ID DB02224), for an inhibition test against SeSSB. Quercetin and taxifolin are structurally similar plant polyphenols [59]. As compared to quercetin (Figure 4A), taxifolin (Figure 4B) is also known as dihydroquercetin, which does not possess the double bond on the ring C (Figure 4B). EMSA was used to analyze the inhibitory effect of taxifolin on the SeSSB. The ssDNA dT35 was biotinylated at the 3′ terminal and incubated with purified SeSSB of different concentrations. The biotin-labeled dT35 could be detected by the streptavidin–horseradish peroxidase conjugate. As shown in Figure 4C, 310 nM SeSSB was sufficient to reach 100% binding of dT35. Through the titration curve (Figure 4D), the dissociation constant of the SeSSB to bind ssDNA dT35 was calculated to be 230 ± 20 nM. To analyze whether taxifolin inhibits the ssDNA-binding activity of the SeSSB, taxifolin (5–400 μM) was included in the binding assay. We found that taxifolin could inhibit SeSSB binding to dT35 (Figure 4E), while quercetin could not (Figure 4F). According to the titration curve, the IC_50_ value of the SeSSB for taxifolin was determined to be 98 ± 12 μM (Figure 4G). Thus, the structure of the ring C, as compared between taxifolin and quercetin, was an important factor for the flavonol inhibition specificity.

### 2.6. Proposed Inhibition Mode of Taxifolin against SeSSB

**Figure 5:**
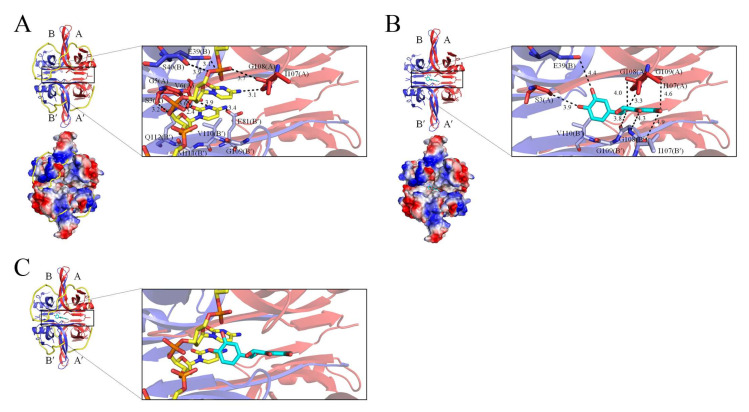
Proposed inhibition mode of taxifolin against SeSSB. (**A**) The ssDNA binding mode of SeSSB. Based on the structural resemblance between SeSSB and EcSSB, their ssDNA-binding modes may be similar. The ssDNA generated from the ssDNA–EcSSB complex (PDB ID 1EYG) is shown in gold in the SeSSB–ssDNA complex. Superposition analysis indicates that residues S3(A), G5(A), V6(A), I107(A), G108(A), E39(B), S40(B), E81(B′), G109(B′), V110(B′), M111(B′), and Q112(B′) might be involved in ssDNA binding at the dimer–dimer interface of the SeSSB. (**B**) The proposed taxifolin binding site. Because of the similarity, taxifolin might bind a site similar to that of quercetin. We manually constructed a binding model of taxifolin to the SeSSB by superimposing the SeSSB structure with the quercetin–PaSSB complex, in which quercetin was replaced by taxifolin. Quercetin and taxifolin might bind to the SeSSB at a similar site, but their binding poses were different. Based on this proposed model, residues S3(A), I107(A), G108(A), G109(A), E39(B), I107(B′), G108(B′), G109(B′), and V110(B′) within the contact distance (<5 Å) might be involved in taxifolin binding. (**C**) The proposed inhibition mode. Superimposing the modeled structures of the ssDNA-bound state and the taxifolin-bound state of SeSSB revealed that residues S3, E39, I107, G108, G109, and V110 were important for both taxifolin (cyan) and ssDNA (gold). Possibly, taxifolin interfered with the SeSSB–ssDNA interaction by occupying the binding site, thus preventing the ssDNA from wrapping fully in the SeSSB and inhibiting the binding activity.

The ssDNA wraps around SSBs via stacking and electrostatic interactions [10]. The EcSSB–ssDNA complex structure revealed the ssDNA-binding path [10]. The EcSSB has numerous essential aromatic and basic residues on the surfaces of the EcSSB tetramer that create a binding path to accommodate ssDNA binding. These ssDNA-binding residues in the EcSSB are conserved in the SeSSB and their ssDNA-binding modes may be similar (Figure 5A). The binding of taxifolin might prevent the ssDNA wrapping and binding of the SeSSB. Previously, the crystal structure of PaSSB complexed with the flavonol inhibitor revealed that a myricetin molecule could be found in a cavity created at the dimer–dimer interface of PaSSB [32]. Complexed crystal structures have revealed that quercetin [31] and myricetin [32] can bind to the PaSSB at a similar site, but their binding poses were different [31]. We propose that taxifolin might bind a site similar to that of quercetin (Figure 5B) and interfere with the SeSSB–ssDNA interaction by occupying the binding site, thus preventing the ssDNA from wrapping fully in the SeSSB and inhibiting the binding activity (Figure 5C). Thus, we manually constructed a binding model of taxifolin to the SeSSB by superimposing the SeSSB structure (determined in this study) with the quercetin–PaSSB complex, in which quercetin was replaced by taxifolin. Based on this proposed model, residues S3(A), I107(A), G108(A), G109(A), E39(B), I107(B′), G108(B′), G109(B′), and V110(B′) within the contact distance (<5 Å) might be involved in taxifolin binding (Figure 5B). Superimposing the modeled structures of the ssDNA-bound state and the taxifolin-bound state of SeSSB revealed that residues S3, E39, I107, G108, G109, and V110 were important for both taxifolin and ssDNA. Accordingly, the binding of taxifolin might interfere with the SeSSB–ssDNA interaction. However, this speculation must be confirmed by further structural and biochemical experiments. Currently, our laboratory is attempting to obtain crystals of the taxifolin–SeSSB complex for the determination of the accurate binding site.

### 2.7. The Taxifolin Structural Interactome

**Figure 6:**
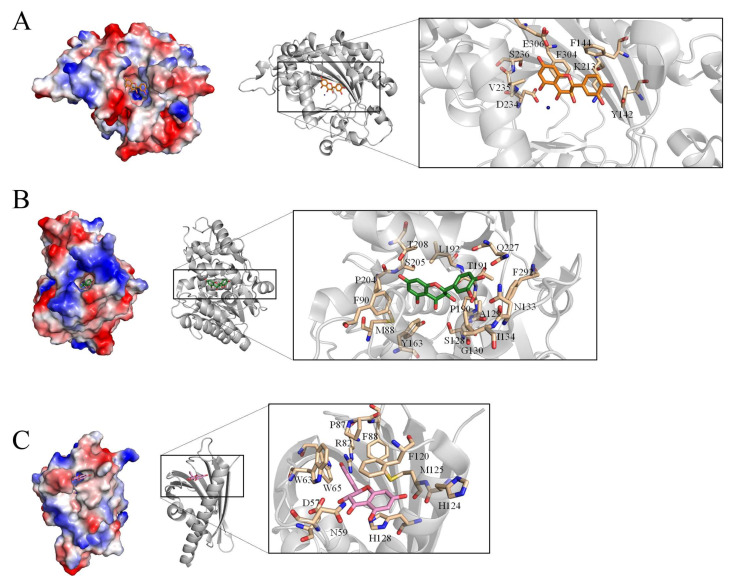
The taxifolin structural interactome. Three taxifolin-complexed protein structures are available in the PDB: (**A**) anthocyanidin synthase (PDB ID 1GP5), (**B**) dihydroflavonol reductase (PDB ID 2C29), and (**C**) WhiE aromatase/cyclase (PDB ID 3TVQ). Although these enzymes are all involved in the biosynthesis of flavonoids, their taxifolin-binding modes are significantly different.

We noticed that there are three taxifolin-complexed protein structures available in the PDB: anthocyanidin synthase (PDB ID 1GP5), dihydroflavonol reductase (PDB ID 2C29), and WhiE aromatase/cyclase (PDB ID 3TVQ). These enzymes are involved in the biosynthesis of flavonoids. They bind taxifolin via different binding environments. For example, anthocyanidin synthase binds taxifolin via residues Y142, F144, K213, D234, V235, S236, F304, and E306 (Figure 6A). Dihydroflavonol reductase binds taxifolin via residues M88, F90, S128, A129, G130, N133, I134, Y163, P190, T191, L192, P204, S205, T208, Q227, and F292 (Figure 6B). WhiE aromatase/cyclase binds taxifolin via residues D57, N59, W63, W65, R82, P87, F88, F120, H124, M125, and H128 (Figure 6C). Given that taxifolin is an important natural product and is being considered for anticancer chemotherapies, further structural studies are needed to understand taxifolin-binding mechanisms for building the whole structural interactome for use in detailed pharmacokinetics and toxicity analyses.

## 3. Discussion

**Figure 7:**
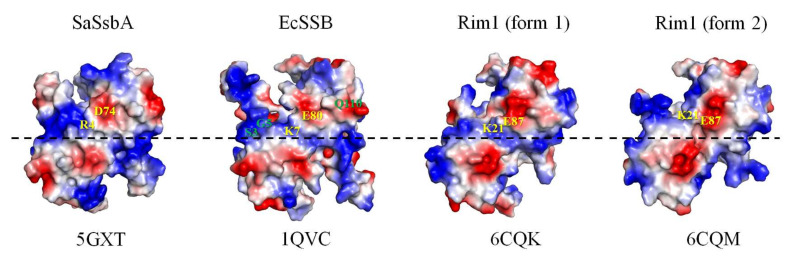
The electrostatic potential comparison of the dimer-dimer interfaces of SaSsbA, EcSSB, and Rim1. The important pair of charged residues (K7/E80) forming a cluster of intermolecular salt bridges at the tetramer formation surface in EcSSB is shown. This pair was also found in SeSSB, but the distance of these corresponding residues in SeSSB was too far (>4.5 Å) to efficiently interact with each other. Rim1 (the form 2) is also present in the case of SeSSB. Residues crucial for tetramerization in SeSSB are colored in green. The pair of charged residues is in gold.

In this study, we determined the crystal structure and examined the size of the ssDNA-binding site of SSB from *S. enterica* serovar Typhimurium LT2, which is a ubiquitous opportunistic pathogen that is highly resistant to antibiotics and the leading cause of human gastroenteritis, and it has also been used in generating a mouse model of human typhoid fever [25]. The crystal structure was solved at a resolution of 2.8 Å, indicating that the SeSSB monomer possesses an OB-fold domain with an additional β6 strand at its N-terminus (Figure 1) and a flexible tail at its C-terminus, as in the EcSSB [21]. The crystal of SeSSB contained two monomers per asymmetric unit, which may indicate the formation of a dimer. However, the gel-filtration chromatography analysis further showed that SeSSB forms a tetramer in solution (Figure 2). The structural and sequence analysis indicated that the tetramer formation mechanisms are different among the SeSSB (this study), ScSsbB [18], SaSsbA [15], SaSsbB [12,14], and SaSsbC [13]. The residues S3 and G5, crucial for tetramer formation in SeSSB, are not conserved in these Gram-positive bacterial SSBs (Table 2). In addition, the residue S3 is also not found in PaSSB, the Gram-negative bacterial SSB [17]. Interestingly, the important pair of charged residues (K7/E80) forming a cluster of intermolecular salt bridges at the tetramer formation surface in EcSSB (Figure 7) was also found in SeSSB (Table 2). However, the distance of these corresponding residues in SeSSB was too far (> 4.5 Å) to efficiently interact with each other. The mitochondrial SSB from *Saccharomyces cerevisiae*, Rim1, also has the pair (K21/E87) at the tetramer formation surface (Table 2 and Figure 7), but Rim1 does not form stable homotetramers and binds DNA as a dimer of dimers [60]. It is worth determining the crystal structures of different SSBs for deeper structural comparisons.

The EMSA results indicate that the SeSSB binds to ssDNA dA35 and dA40 to form a complex in which a single tetramer is bound to the ssDNA (Figure 3). Two SeSSB tetramers were bound to dA45, dA50, and dA55. Based on these EMSA patterns, the apparent binding-site size (stoichiometry) of the SeSSB determined by using such a series of ssDNA dA homopolymers was approximately 22 nt. Similarly, the apparent binding-site size of the SeSSB was also 22 nt when using a series of ssDNA dT homopolymers for determination [48,49]. Thus, the base preference was not the determinant for the estimation of the ssDNA-binding-site size of the SeSSB.

We previously estimated the ssDNA binding-site size of the SeSSB, KpSSB [56], and PaSSB [57] to be 22, 26, and 29 nt per tetramer, respectively, using a series of ssDNA dT homopolymers through EMSA. Although these SSBs share a similar ssDNA-binding domain, they bind to ssDNA with different stoichiometries. We noted that the genomic DNA lengths of these bacteria are significantly different. The genomic DNA lengths of the *S. enterica* [25], *K. pneumonia* [61], and *P. aeruginosa* [62] are 4.8, 5.3, and 6.3 million base pairs, respectively. Namely, the length followed the order: *P. aeruginosa* > *K. pneumonia* > *S. enterica*. The relationship we found was that the longer the genomic DNA length of the bacterium, the bigger the binding-site size of the SSB. Given that bacteria have varying genomic DNA sizes, their SSBs may need to evolve gradually to have different binding-site sizes to better coordinate DNA metabolism in each bacterium. However, this speculation regarding the genomic DNA length–binding-site size relationship should be confirmed experimentally.

**Figure 8:**
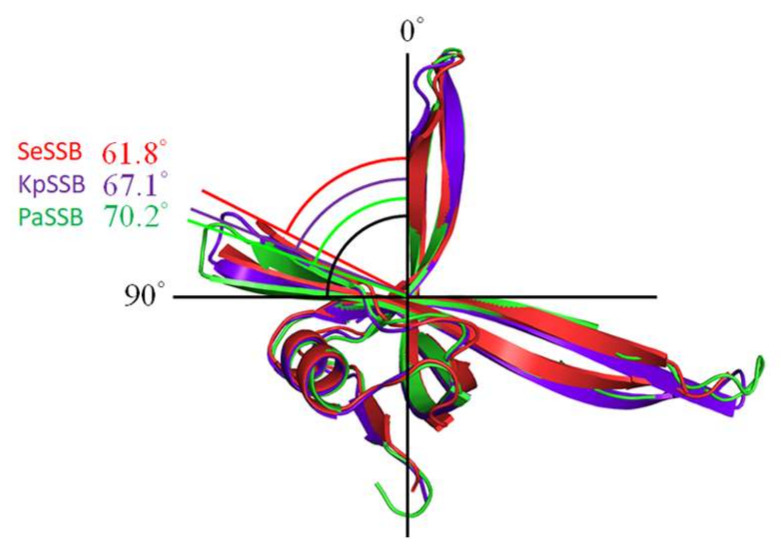
Structural differences among SeSSB, KpSSB, and PaSSB. The ssDNA interaction cavity of SSB is created by strands β1′ and β4. The angles between strands β1′ and β4 of the SeSSB, KpSSB, and PaSSB were 61.8°, 67.1°, and 70.2°, respectively. That is, the wider the groove of the OB fold, the bigger the binding-site size of the SSB.

Despite the OB folds having a similar appearance, we noted that the sizes of the ssDNA-binding groove in the SeSSB, KpSSB, and PaSSB were somehow a little different. Structurally, the angles between strands β1′ and β4 of the SeSSB, KpSSB, and PaSSB were 61.8°, 67.1°, and 70.2°, respectively (Figure 8). That is, the wider the groove of the OB fold, the bigger the binding-site size of the SSB. This structure–function relationship might explain why their ssDNA binding-site sizes are distinct.

Taxifolin, a unique bioactive flavonoid, has a wide range of biological activities and pharmaceutical relevance against inflammation, malignancies, microbial infection, oxidative stress, cardiovascular disease, and liver disease [34]. For the first time, we identified that taxifolin can inhibit the activity of an OB-fold protein, namely, the SeSSB (Figure 4). Almost all OB-fold proteins are widely associated with binding to a variety of DNA substrates and play essential roles in both prokaryotic and eukaryotic cells by participating in DNA metabolism, including replication, repair, recombination, and replication fork restart [11]. Taxifolin possibly binds to these proteins and produces some cellular signaling pathways. Whether some of the broad biological activities of taxifolin are based on the inhibition against certain OB-fold proteins, i.e., not only SSBs, remains to be experimentally demonstrated.

The development of clinically useful small-molecule antibiotics has been a seminal event in the field of infectious diseases [63,64,65]. Flavonols are safe as pharmaceuticals because they have few side effects in human use [66]. The potential of flavonoids for use in antibacterial chemotherapy has been essentially confirmed [36,67,68]. For example, the flavonoids myricetin, taxifolin, kaempferol, and luteolin in *Mandragora autumnalis* are known to significantly inhibit the growth of many bacterial and fungal strains and show the greatest antibacterial activity against the *K. pneumoniae* strain [69]. The antibacterial mechanisms of flavonoids are summarized as follows: inhibition of nucleic acid synthesis, inhibition of cytoplasmic membrane function, inhibition of energy metabolism, inhibition of the attachment and biofilm formation, inhibition of the porin on the cell membrane, alteration of the membrane permeability, and attenuation of the pathogenicity [68]. Nucleic acid metabolism is one of the most basic biological functions, and, thus, bacterial SSBs should be a prime target in antibiotic development [70,71]. Myricetin [31,32] and taxifolin (Figure 4), but not quercetin, were found to be inhibitors against SSBs. Although they might bind to the SeSSB at a similar site (Figure 5), their binding modes, especially the binding poses to SSBs, could be different [31].

In conclusion, the crystal structure of the SSB from *S. enterica* serovar Typhimurium, a leading cause of human gastroenteritis [25], provided a molecular insight into the basis of drug development. The cavity at the dimer–dimer interface of the SSB (Figure 5) could be a suitable target for inhibitor design. Taxifolin, a naturally occurring product with potent anticancer activities, was also capable of inhibiting SeSSB activity. The more complexed structures still need to be solved to extend the taxifolin interactome for further clinical use (Figure 6).

## 4. Materials and Methods

### 4.1. Protein Expression and Purification

Construction of the SeSSB expression plasmid has been reported [49]. The expression vector pET21b-SeSSB was transformed into *E. coli* BL21 (DE3) cells and grown in LB medium at 37 °C. The overexpression was induced by incubating with 1mM isopropyl thiogalactopyranoside for 9 h at 25 °C. The SeSSB protein was purified from the soluble supernatant by Ni^2+^-affinity chromatography (HisTrap HP; GE Healthcare Bio-Sciences, Piscataway, NJ, USA), eluted with Buffer A (20 mM Tris-HCl, 200 mM imidazole, and 0.5 M NaCl, pH 7.9), and dialyzed against a dialysis buffer (20 mM HEPES and 100 mM NaCl, pH 7.0; Buffer B). Protein purity remained at >97% as determined by SDS-PAGE (Mini-PROTEAN Tetra System; Bio-Rad, Hercules, CA, USA).

### 4.2. Crystallography

Purified SeSSB was concentrated to 16 mg/mL for crystallization. Commercially available screens from Hampton research and Jena biosciences were employed for the crystallization trials. Crystals of the SeSSB were grown at room temperature by hanging drop vapor diffusion in 15% PEG400 and 100 mM MES at pH 6.5. Data were collected using an ADSC Quantum-315r CCD area detector at SPXF beamline BL13C1 at NSRRC (Taiwan). All data integration and scaling was carried out using HKL-2000 [72]. There were two SeSSB monomers per asymmetric unit. The crystal structure of SeSSB was solved at 2.87 Å resolution with the molecular replacement software Phaser-MR [73] using EcSSB as model (PDB ID 1EYG). A model was built and refined with PHENIX [74] and Coot [75]. The final structure was refined to an *R*-factor of 0.253 and an *R*_free_ of 0.284 (Table 1). Atomic coordinates and related structure factors have been deposited in the PDB with accession code 7F25.

### 4.3. Gel-Filtration Chromatography

Gel-filtration chromatography was carried out by the AKTA-FPLC system. In brief, purified SeSSB (5 mg/mL) in Buffer C (100 mM NaCl and 100 mM MES, pH 6.5) was applied to a Superdex 200 prep-grade column (GE Healthcare Bio-Sciences, Piscataway, NJ, USA) equilibrated with the same buffer. The column was operated at a flow rate of 0.5 mL/min, and 0.5 mL fractions were collected. The proteins were detected by measuring the absorbance at 280 nm. The column was calibrated with proteins of known molecular weight: thyroglobulin (670 kDa), γ-globulin (158 kDa), ovalbumin (44 kDa), and myoglobin (17 kDa). The *K*_av_ values for the standard proteins and the SeSSB protein were calculated from the equation *K*_av_ = (*V*_e_ − *V*_o_)/(*V*_c_ − *V*_o_), where *V*_o_ is column void volume, *V*_e_ is elution volume, and *V*_c_ is geometric column volume.

### 4.4. EMSA for Determining the ssDNA Binding-Site Size

Various lengths of ssDNA dA homopolymers were custom synthesized. Radiolabeling was carried out with [γ^32^P]ATP (6000 Ci/mmol) and T4 polynucleotide kinase (Promega, Madison, WI, USA). The SeSSB protein (0, 500, 1000, 2000, and 4000 nM) was incubated for 30 min at 25 °C with 1.7 nM DNA substrates (dA35–55) in a total volume of 10 μL in 20 mM Tris–HCl pH 8.0 and 100 mM NaCl. Aliquots (5 μL) were removed from each reaction solution and added to 2 μL of gel-loading solution (0.25% bromophenol blue and 40% sucrose). The resulting samples were resolved on a native 8% polyacrylamide gel at 4 °C in TBE buffer (89 mM Tris borate and 1 mM EDTA) for 1 h (for dA30 and dA35) or 1.5 h (dA45–55) at 100 V and visualized by autoradiography.

### 4.5. EMSA for Inhibition Assay

EMSA for an inhibition test against SeSSB was conducted in accordance with a previously described protocol [76,77]. The 5′-biotinylated oligonucleotide (dT35) was synthesized for this inhibition assay. The final concentration of the labeled oligonucleotide was 30 fmol/μL. EMSA was performed using LightShift Chemiluminescent EMSA Kit (Thermo Scientific, USA) with a minor modification for SeSSB. In brief, SeSSB (0–2500 nM) was incubated for 60 m at 37 °C with DNA substrate (30 fmol/μL) in a total volume of 6 μL in 40 mM Tris–HCl (pH 7.5) and 50 mM NaCl. Following incubation, 4 μL of a dye mixture (0.01% bromophenol blue and 40% glycerol) was added. Native polyacrylamide gel (8%) was pre-electrophoresed at 110 V for 10 min. Thereafter, the resulting samples were loaded and resolved on pre-run gel and electrophoresed at 100 V for 1 h in TBE running buffer (89 mM Tris borate and 1 mM EDTA). The protein–DNA complexes were electro-blotted to positively charged nylon membrane (GE, USA) at 100V for 30 min in fresh and cold TBE buffer. Transferred DNA was cross-linked with nylon membrane using a UV-light cross-linker instrument equipped with 312 nm bulbs for 10 min exposure. Biotin-labeled DNA was detected using streptavidin–horseradish peroxidase conjugate and chemiluminescent substrate contained in SuperSignal™ West Atto Ultimate Sensitivity Substrate (Pierce Biotechnology, Waltham, MA, USA). The ssDNA-binding ability of the protein was estimated through linear interpolation from the concentration of the protein that bound 50% of the input DNA. To assess whether taxifolin inhibits the binding activity of SSB, SeSSB (320 nM) with DNA substrate was incubated with taxifolin (0, 5, 20, 40, 60, 80, 100, 200, and 400 μM) for 60 m at 37 °C. The resultant protein solution was then analyzed using EMSA. Quercetin (0, 5, 20, 40, 60, 80, 100, 200, and 400 μM) was also used for this inhibition test.

## Figures and Tables

**Table 1:** Data collection and refinement statistics.

Data Collection	
Crystal	SeSSB
Wavelength (Å)	0.975
Resolution (Å)	28.5–2.87
Space group	P3_2_21
Cell dimension	
*a*, *b*, *c* (Å)	91.89, 91.89, 61.05
*α, β,γ* (°)	90, 90, 120
Redundancy	5.3 (4.9)
Completeness (%)	99.9 (99.7)
<I/σI>	20.3 (2.3)
CC_1/2_	0.980 (0.918)
Refinement	
No. reflections	7050
*R*_work_/*R*_free_	0.253/0.284
No. atoms	
Protein	212
Water	1
*r.m.s.* deviations	
Bond lengths (Å)	0.011
Bond angles (°)	1.51
Ramachandran plot	
Favored (%)	98.00
Allowed (%)	2.00
Outliers (%)	0
PDB ID	7F25

Values in parentheses are for the highest resolution shell. CC_1/2_ is the percentage of correlation between intensities of random half-data sets.

**Table 2:** The corresponding residues crucial for tetramerization of SSB.

SeSSB	EcSSB	SaSsbA	Rim1
S3	S3	None	K16
G5	G5	None	D18
Q111	Q110	E104	N114
K7	K7	R4	K21
E80	E80	D74	E87

Hydrogen bonds and salt bridges were formed at the dimer–dimer interface of the SeSSB: S3(A)–Q111(B′), G5(A)–Q111(B′), G5(B)–Q111(A′), Q111(A)–G5(B′), Q111(B)–S3(A′), and Q111(B)–G5(A′). These residues were also conserved in many Gram-negative bacterial SSBs, such as EcSSB. The distance of the important pair of charged residues (K7/E80) in SeSSB was too far (>4.5 Å) to efficiently interact with each other.

## Data Availability

Atomic coordinates and related structure factors were deposited in the PDB with accession code 7F25.
